# Adaption, implementation and evaluation of collaborative service improvements in the testing and result communication process in primary care from patient and staff perspectives: a qualitative study

**DOI:** 10.1186/s12913-017-2566-8

**Published:** 2017-08-30

**Authors:** Ian J. Litchfield, Louise M. Bentham, Richard J. Lilford, Richard J. McManus, Ann Hill, Sheila Greenfield

**Affiliations:** 1 Institute of Applied Health Research, College of Medical and Dental Sciences, University of Birmingham, Edgbaston, Birmingham, B15 2TT UK; 2 WMS – Population Evidence and Technologies, University of Warwick, Coventry, UK; 3 National Institute for Health Research (NIHR) School for Primary Care Research, Nuffield Department of Primary Care Health Sciences, University of Oxford, Oxford, UK; 4 Head of Transformation, Worcestershire Acute Hospitals NHS Trust, Worcester, UK

**Keywords:** Clinician-patient communication/relationship, Healthcare delivery/health services research, Cllaborative/interdisciplinary care

## Abstract

**Background:**

Increasing numbers of blood tests are being ordered in primary care settings and the swift and accurate communication of test results is central to providing high quality care. The process of testing and result communication is complex and reliant on the coordinated actions of care providers, external groups in laboratory and hospital settings, and patients. This fragmentation leaves it vulnerable to error and the need to improve an apparently fallible system is apparent. However, primary care is complex and does not necessarily adopt change in a linear and prescribed manner influenced by a range of factors relating to practice staff, patients and organisational factors. To account for these competing perspectives, we worked in conjunction with both staff and patients to develop and implement strategies intended to improve patient satisfaction and increase efficiency of existing processes.

**Methods:**

The study applied the principles of ‘experience-based co-design’ to identify key areas of weakness and source proposals for change from staff and patients. The study was undertaken within two primary practices situated in South Birmingham (UK) of contrasting size and socio-economic environment. Senior practice staff were involved in the refinement of the interventions for introduction. We conducted focus groups singly constituted of staff and patients at each practice to determine suitability, applicability and desirability alongside the practical implications of their introduction.

**Results:**

At each practice four of the six proposals for change were implemented these were increased access to phlebotomy, improved receptionist training, proactive communication of results, and increased patient awareness of the tests ordered and the means of their communication. All were received favourably by both patients and staff. The remaining issues around the management of telephone calls and the introduction of electronic alerts for missing results were not addressed due to constraints of time and available resources.

**Conclusions:**

Approaches to tackling the same area of weakness differed at practices and was determined by individual staff attitudes and by organisational and patient characteristics. The long-term impact of the changes requires further quantitative evaluation.

## Background

The number of blood tests ordered in primary care continues to rise and the rapid and precise communication of blood test results remains central to ensuring patients receive timely and appropriate care [1]. However, the process of testing and result communication is complex and reliant on the coordinated actions of care providers, external groups in laboratory and hospital settings, and patients. This fragmentation leaves it vulnerable to error from a number of social and organisational factors [2, 3]. Where errors do occur, they have implications for both patient safety and satisfaction [1, 3–9] and medico-legal concerns for health-care providers [10–12].

The need to improve an apparently fallible system is clear [4–7] and yet primary care is a complex, adaptive system that does not necessarily adopt change in a linear and prescribed manner [13–15]. Instead the improvement of any aspect of the service is an intricate process influenced by a number of interrelated factors, including readiness for change [16], motivational levels of staff [17, 18], and contextual factors such as organisational climate [19]. Practices are already stretched by the need to incorporate the competing demands of growing workloads and decreasing resources [20] and so engaging staff in change can be difficult where, as in the instance of testing and result communication, formal requirements or guidelines are lacking [21, 22].

In the UK, the Test Result Communication Knowledge, Evaluation and Development (TRaCKED) study used the principles of ‘Experience-based Co-design’ [23] to work with patients and staff to improve the testing and result communication process for clinical investigations in primary care. In doing so we identified six key areas of weakness in the existing process that ranged from a delay in accessing phlebotomy to the lack of patient awareness of how to retrieve results [24]. A number of ideas for improvement were sourced from patients and staff that attempted to reconcile the preferences of both groups with the available resources and we aimed to refine, implement and evaluate these proposals [6, 7]. Here we report on the factors that influenced the practice decisions on which issues should be dealt with and how, and then we describe the post-implementation evaluation of these interventions from the perspectives of both staff and patients.

## Methods

### The TRaCKED study

**Fig. 1:**
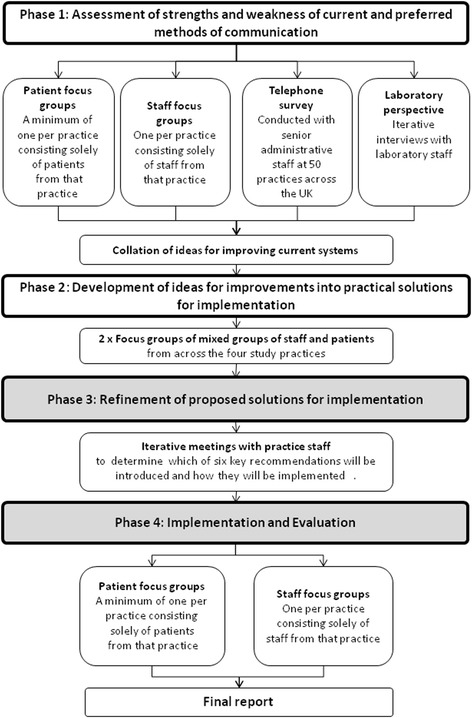
The four phase design of the TRaCKED Study

The TRaCKED study worked closely with primary care practices within South Birmingham to improve the testing and result communication process and consisted of four phases. The first phase aimed to understand the strengths and weaknesses of the current testing and result communication process; the second to develop ideas to improve any identified weaknesses; the third to refine these ideas so they are applicable to the practices where they were introduced, and the fourth to implement and then evaluate the changes to existing processes with staff and patients (see Fig. 1). Here we present our findings from the third and fourth phases.

### Settings

The four practices purposively selected for TRaCKED reflected a range of size and socio-economic environment, with an apparent range of pathways for communicating results, encompassing a number of overlapping methods and systems [25]. The study practices belonged to Birmingham Crosscity Commissioning Group (BXCCG). These commissioning groups are clinically-led statutory NHS bodies responsible for the planning and commissioning of health care services for their local area. BXCCG is the fourth largest of all clinical commissioning groups in England with 95 member practices, an annual budget of £1 billion and commissioning services for a population of around 710,000. All four practices were invited to take part in the final two phases of the study and Practices 1 and 2 decided to participate (see Table 1). Each practice used a range of clinical and non-clinical staff involved in the result communication process including receptionists, phlebotomists, health care assistants, nurses, and family practitioners [6].

**Table 1:** Characteristics of the TRaCKED study practices

General practice study ID	Number of patients registered	Number of full time equivalent general practitioners (GPs)	IMD code ranking^a^	Clinical management system
Practice 1	23,727	7.3	15,066	SystmOne
Practice 2	7059	6.3	871	EMIS
Practice 3	5914	3.0	13,866	EMIS
Practice 4	27,430	12.3	8447	EMIS

^a^Index of multiple deprivation (IMD) ranking out of 32,482 LSOAs (lower super output levels) in England. The IMD codes, produced by the UK government and first released in 2004 and updated 2010, provide indicators of deprivation in local authority areas to inform health and social policy, the lower the score the more deprived the area [24]

### Phase 3: Selection and refinement of proposed solutions

In Phases 1 and 2 of the TRaCKED study, six key areas of weakness in the current test result communication process were identified (see Fig. 1) [24]. In Phase 3 we asked the two participating practices to consider these and make suggestions as to how these could be improved and to select those which could be implemented in their own practice. The six areas of weakness identified were: delay in securing phlebotomy appointments; the lack of an electronic alert for practice staff triggered by delayed or absent results, lengthy waits for patients asked to telephone for results; receptionists being asked to communicate clinical information to patients; the absence of any proactive communication of results; and poor patient awareness of the tests ordered and the precise pathway for result communication.

A number of solutions were proposed to strengthen these areas and we worked closely with staff at each of the two practices to determine which particular suggestions for improvement were the most pertinent, acceptable, and logistically feasible. In doing so we carried out three group meetings at each practice over the course of six months. The first was conducted with a cross-section of clinical and non-clinical staff. At Practice 1 these consisted of a number of Family Practitioners (FPs), and nurses; at Practice 2 FPs were joined by a lead receptionist and a practice manager. Each practice then formed a ‘working group’ of 2 key individuals that were present at the initial Phase 3 meeting. These individuals would be responsible for the final form and implementation of the amendments to existing systems. At Practice 1 the members consisted of two senior FPs and at Practice 2, the practice manger and a senior FP partner (see Table 2). At the initial Phase 3 meeting all six of the proposed solutions were explored in turn and a decision made as to which would be adopted. The second and third meetings focussed on the operational aspects of introducing these interventions to determine the exact form the amendment would take and the proposed timescale of adoption. At each of the Phase 3 meetings the research team was represented by IL and all were digitally recorded and transcribed verbatim.

**Table 2:** Schedule and personnel for Phase 3 meetings

	Practice 1	Practice 2
Initial Phase 3 meeting	GP ×6 Nurses ×2	GP × 2 Lead receptionist ×1 Practice Manager X1
Working group meeting 1	GP ×2	GP ×1 PM × 1
Working group meeting 2	GP ×2	GP ×1 PM × 1

### Phase 4: Evaluation phase

To evaluate the changes practices had chosen to make to their current systems, we conducted two focus groups at each practice 3 months after implementation of the changes: one with staff and the other with patients.

#### Focus groups

Each focus group was practice-specific and conducted separately with either staff or patients. At both practices, staff meetings were arranged by the practice manager who invited all staff currently playing a role in the testing and result communication process, namely FPs, Practice Nurses (PNs), Health Care Assistants (HCAs), Receptionists, and Practice Managers (PMs).

The patient members of each focus group were drawn from their practice patient participation group and invited via their respective chair. The topic guide at each meeting was specific to that practice and based upon the amendments selected for implementation during Phase 3. At each we asked participants to describe their attitudes toward, and experiences of the changes implemented at that practice, discussing any perceived strengths, weaknesses and how they might be further refined to improve their sustainability. Every focus group was moderated by IJL and they were digitally recorded and transcribed verbatim.

### Analysis

Each transcript was examined closely and the findings analysed using a framework analysis [26] by IJL (research fellow with a background in occupational medicine), SMG (professor of medical sociology) and LMB (a senior research nurse), who met and agreed on emerging themes to decide on a coding framework.

## Results

### Phase 3: Selection and refinement of proposed solutions

A number of suggestions for improvement in each of the 6 identified areas were made (see Table 3). Over the course of three Working Group meetings held with each practice, these suggestions for change previously identified were either dropped or refined depending on which changes practices chose to adopt. In summary, both practices chose to 1) Increase access to phlebotomy, 2) Improve receptionist training, 3) Introduce proactive communication of results and 4) Increase patient awareness of the tests ordered and the means of communication. Neither chose to address 5) Improved management of phone calls from patients seeking results nor 6) Creation of an alert for missed or delayed results though we understand that EMIS web has since included that functionality [27].

**Table 3:** Practice characteristics showing suggested solutions and those adopted

Issue	Proposed solutions	Solution (those implemented in italics)	
	Practice 1 and 2	Practice 1	Practice 2
1. Delay in access to phlebotomy	1) Reconfigure appointments to meet demand. 2) Increase the hours of phlebotomists. 3) Train existing staff in phlebotomy to provide support for phlebotomists.	*Employed additional HCA* *Extended clinic to the afternoon*	*Trained existing staff in phlebotomy.* *Kept additional appointments free*
2. Receptionists reporting clinical information	1) Support data protection act compliance by receptionists 2) Training for receptionists in how to communicate potentially sensitive information 3) Improving access to teleconsultations with GPs 4) Greater clarity in the script provided by GPs	*The importance of the accurate communication of result information was raised with reception staff.*	*Receptionists instructed to advise patients that there may be results outstanding.* *The number of teleconsultations with GPs was increased.*
3. Lack of routine communication of results	1) via SMS 2) via letter 3) via email	*Selective use of SMS.*	*Selective use of SMS.* *Letters routinely sent to patients with abnormal results advising them to book/keep their appointment.*
4. Lack of patient awareness of the communication pathway	1) Poster on wall in waiting room 2) Information leaflet for patients detailing tests ordered and the means of retrieving their results embedded within clinical management system.	*Information leaflet for patients printed and distributed by GPs.*	Issues with software provider meant it could not be achieved within the timescale of the study.
5. Delays for patients seeking results via telephone	1) Precise time slot for calling for results 2) Separate phone line for results 3) Call waiting	Patient demographic deemed unsuitable.	New telephone system recently procured.
6. Lack of an alert for delayed or missing results	Alerts embedded in the clinical management system issued if: 1) The result has not been returned by the laboratory 2)The result has not been seen by GPs 3) The result has reached patients	The technical development needed could not be achieved within the time scale.	Would not commit to improving existing system with laboratory services out for tender

In the following section we present staff attitudes to the proposed solutions and the factors that influenced which issues they chose to address.

1. Increased access to phlebotomy
  Staff at both practices acknowledged the need to reduce the delay in waiting for a phlebotomy appointment, though each ultimately took different approaches to achieve this. Acknowledging that the demand for phlebotomy meant nurses would often perform the task, when their time might be used more effectively elsewhere in the practice. Practice 1 employed an additional Health Care Assistant (HCA) to undertake phlebotomy.
  What we still want, as a practice, is for our nurses, who are much better qualified, not to be doing stuff like that – that’s just an expensive use of nurses’ time, so we’re trying to move towards HCAs for as much as we can. – *FP1 (male) Practice 1*
  By employing an additional HCA Practice 1 could run an additional Phlebotomy clinic in the afternoon.
  We’ve just employed a new HCA – virtually full-time – well, it should be about three or four days…we’re doing phlebotomy morning and afternoon, that’s something that’s changed. – *FP1 (male) Practice 1*
  Practice 2 trained existing office staff in phlebotomy to provide cover for clinical staff.
  The receptionist has just finished her competencies; she’s actually going to cover home visits for our HCA when she’s on leave next year which is the first time we’ve ever had that, so yeah, the teams expanded, it’s giving us a bit of flexibility. – *PM (female) Practice 2*
  Practice 2 also increased the number of spare appointments within each phlebotomy clinic to allow for increased same-day access.
  We save appointments for the day so there’s a slot the Doctor can utilise rather than sending people away. – *PM (female) Practice 2*
2. Improved receptionists’ training
  Senior staff at Practice 1 did feel that their new receptionists would benefit from external training.
  We’ve discussed this just this week actually… they don’t have any out of house training. It’s all in-house training. [They’re doing a receptionist training day at the BMI in June] which is a good idea…we’ve got a couple of new receptionists that I think would benefit from that. – *FP1 (male) Practice 1*
  Both FPs in the working group at Practice 1 felt it was important for FPs to be clear in the notes accompanying results which they intend the receptionist to read to patients.
  I think the main thing is to remember to write what you want the receptionist to say to the patient, and actually write the words – then there’s no confusion. – *FP1 (male) Practice 1*
  As partners and doctors, we talked about the importance of having a clear result to communicate to the patient and the staff know that they’re allowed to say what’s written in the box but not anything in brackets that’s for our benefit. That has definitely been reiterated recently. – *FP2 (female) Practice 1*
  During discussions about the targeted training of receptionists used to relay result information, it became apparent that increased pressure on limited practice resources meant opportunities to introduce this training were limited.
  I think there’s definitely room for training; I think it’s just finding time to do it. Honestly, we are just running and tripping over ourselves at the moment and we have been doing now for the last eighteen months, never known anything like it. It started in the new year last year usually we have a lull, and in that lull I do all sorts of things because it’s definitely quieter, we haven’t had that period, we didn’t get it last year, we didn’t get it this year, and I doubt we’re going to get it next. – *PM (female) Practice 2*
  Therefore at Practice 2, instead of concerted training, receptionists were asked to remind patients of their responsibility to ensure that all results have been retrieved.
  Receptionists don’t know if that’s the only result or one of six so our staff are trained to say “Well this result’s back and that particular result is normal but obviously we don’t know how many the doctor ordered”. – *PM (female) Practice 2*
3. Proactive communication of results
  Patients at our study practices expressed a preference for receiving low impact results (i.e. those requiring no further action) via electronic methods such as SMS. Senior staff felt the formal introduction of SMS might jeopardise data protection.
  We felt it’s not a good idea to enact as policy for a lot of reasons to do with confidentiality. If you put your phone down and I text it to you …it’s a visible sort of thing. – *FP2 (female) Practice 1*
  There were also concerns over practice liability if patients failed to receive results.
  Phone numbers can change, and phones can be lost. If you don’t know their phone is lost and you don’t know they’ve not got the message, then that comes back to us. – *FP2 (female) Practice 1*
  The staff at Practice 2 were enthusiastic about the idea of using SMS and could see how it might relieve pressure on busy receptionists.
  It’s a good idea … You could set the system up so that ‘normal - no action’ was an automatic SMS….The more that we can do here without involving receptionists, the receptionists are just… you know it is always chaotic in reception. – *FP (female) Practice 2*
  The practice manager at the same practice was wary of patients not knowing exactly which results to expect; particularly where multiple tests were ordered.
  The patient would need to know which results to expect so that they can tick them off themselves. – *PM (Female) Practice 2*
  The same practice manager felt that in the long-term secure emails may be the way forward.
  It is surprising how many do have email addresses. And email addresses are less likely to change as often as mobile phone numbers. So actually email is probably the better, safer way, than even text messages. – *PM (Female) Practice 2*
  For staff at Practice 2 their involvement in the study led to a raised awareness of potentially serious problems with their current governance of result information.
  We looked at abnormal results…If we had an appointment we didn’t write to them because we just wrote on the results: “Discuss with doctor in appointment”. Patient then with an abnormal B12 did not come up and attend the appointment because they cancelled it, and because they cancelled it, it went off the system and it wasn’t identified as an issue until it was picked up on a medication review nearly a year later. – *FP (female) Practice 2*
  As a result, Practice 2 now systematically issue letters to patients with abnormal results.
  In the letter for patients we are going to say: “If you already have an appointment please keep it”. I know they’ve got an abnormality... needs to be seen...check that they’ve got an appointment. Normally I’d have done nothing; they just don’t know the test was abnormal. It’s going to cost a fortune in writing to people but medico-legally…? – *FP (female) Practice 2*
4. Increasing awareness of the tests ordered and the mean of communication
  A prototype template for a test result information leaflet was developed in a series of discussions with staff at who liked that it emphasised patient responsibility for retrieving results and provided a link for further information about the tests requested.
  I think something to hand to the patient to remind them of their responsibility to ring up for the results is a good idea. – *FP2 (female) Practice 1*
  We need this message: “It’s important that you contact the surgery” and then “See Lab Tests Online for further information on the implications of your test”. So those two are really good. – *FP (female) Practice 2*
  Practice 1 was able to begin piloting the information leaflet for patients.
  You have to click on the form, bring the form up, and then print it, so it’s an extra click, if you like…We have put in the bottom in bold what times to call, and you can put on what day to call as well, which I do routinely. – *FP2 (female) Practice 1*
  Currently the large central laboratory used by both practices operates ‘offline’. However it was acknowledged that in the event test orders are made online patients would still benefit from an information leaflet outlining the tests ordered.
  If we do online lab requests we still need a letter like that, that goes to patients saying: “this is what’s being requested”… – *PM (female) Practice 2*
5. Improved management of telephone calls relating to results
  One of the FPs at Practice 1 felt the patient demographic was unsuitable to adapt to changes in the telephone system and so the introduction of a separate phone number for retrieving results was deemed inappropriate due to the age of patients.
  It’s all old dogs, new tricks. I mean, probably 70-80% of tests we do are on over 70’s and you’re asking them to ring different numbers to the ones they’ve been ringing for 20 years, so it sounds nice when you’ve got sort of young people sitting round the room saying ‘nothing could be simpler’, but then you’ve got an old person, who will still ring the main number, and what do you do? Just say ‘I’m sorry, this is the number…’ and then you’re just irritating people you see? – *FP1 (male) Practice 1*
  At Practice 2, and independent of the project, they had recently introduced a new telecommunication system for managing calls that had improved functionality for handling multiple calls.
  They are queued, but they are not told “you’re fourth in the queue”, I don’t believe so…It’s called Auto Attendant that we have. With the new system, if it’s busy it will tell you it’s busy, which is one step better than before where it just rang and rang and rang. – *PM (female) Practice 2*
6. Creating an alert for delayed or missing results
  Current clinical systems do not possess the functionality to provide alerts for missing or delayed results. That the system for ordering tests was due to go “online” meant that staff at Practice 1 did not want to invest resource amending the existing system.
  I don’t think we should waste a lot of time...[on alerts] – *FP (male) Practice 1*
  Staff at Practice 2 commented that engaging their clinical software provider proved difficult as they relied on a customer poll to decide which additional functionality they would next develop. In the case of result communication, these market driven forces were insufficient for the software provider to address the problem.
  As far as they are concerned it’s pretty bomb-proof, the front end, getting to the lab… The back end may have a bit of an issue but they won’t appreciate that the way we have, as we have reviewed it.…they’ve not been helpful EMIS, they said to me “OK, you want a change making; you’ve got to put it on the list [for voting]”. – *FP (female) Practice 2*
  The Practice Manager at Practice 2 felt that greater openness in sharing problems and solutions would be an important starting point to improving result communication more broadly.
  It’s an openness really that we need to look into ‘cause how many times actually in these practices has it gone wrong? We don’t know because people don’t actually share this sort of thing… – *PM (female) Practice 2*

### Evaluation phase

#### Focus groups

Each solution for change was introduced at a different time at each practice. In order to provide the greatest opportunity for staff and patients to have experienced them the two focus groups convened at each practice were conducted three months after the last changes were implemented. At each practice one group consisted of staff, the other of patients. Staff focus groups were attended by both clinical and non-clinical staff with direct experience of at least one of the changes implemented. The staff meeting at Practice 1 was attended by a large number of FPs, one of whom was a member of the working group from Phase 3 the other FP member having since retired. The focus group at Practice 2 was attended by the two members of the working group from the previous phase in addition to the office manager (see Table 4). The patient groups were drawn from each practices’ Patient Participation Group which had been established for a longer period of time at Practice 1.

**Table 4:** Characteristics of staff and patient focus groups for the evaluation phase

	Practice 1 (P1)	Practice 2 (P2)
Attendees staff focus group		
General Practitioners (GP)	6	1
Practice managers (PM)	0	1
Practice nurses (PN)	2	0
Administrative staff (AS)	1	1
Attendees patient focus group		
Female patients	3	2
Male patients	4	2

In the following section we describe in turn staff and patient experiences of the various solutions introduced at each practice.

1. Increased access to phlebotomy
  Practice 1 chose to employ an additional HCA to improve accessibility and reported that as a result wait times had been reduced.
  Yeah, we’ve had quite an increase in phlebotomy [capacity] ‘cause at one stage we were up to two plus weeks wait… now the access to phlebotomy is better, [the nurses] can be freed up to do what they’re meant to do – which isn’t taking blood. – *FP1 (male) Practice 1*
  A patient at Practice 1 commented on the improved accessibility to phlebotomy.
  I’ve had recent tests and I got an appointment the exact day I wanted and the exact time I wanted it. – *Patient 1 (male) Practice 1*
  Practice 2 sought to provide extra capacity during busy periods by training existing administrative staff in phlebotomy to provide cover.
  We now have people we pay to be phlebotomists…two reception staff who step in if we’re overloaded or if someone’s on holiday. – *PM (female) Practice 2*
  In addition Practice 2 increased the number of empty phlebotomy slots so that patients who need phlebotomy on the day can have that access which had been utilised by one of their patients.
  Like with Mum, the doctor would say, “Go down and request an appointment for today” and then I would know that I can go to the desk and say, “The doctor’s just told me ... [to have it done to today]” and they will slot you in! – *Patient 3 (female) Practice 2*
  The point was made by a FP at Practice 2 that no matter how many appointment slots might be available, there is still a degree of flexibility required of patients.
  Often it will be next day but I think it depends on how flexible they are…if they say, “I can only come in on Thursday at 3 o’clock”, well... – *FP (female) Practice 2*
2. Improved Receptionists’ training
*Training receptionists to acknowledge the potential of multiple test results*
    Receptionists at both practices are now instructed to advise patients there may be further results yet to be returned by the laboratory or processed by practice clinicians. At Practice 1, patients were impressed with their recent experience of receptionists communicating results.
    Phoned the receptionist. Good as gold: “Sorry, you’ve got one result back which is fine, the rest of your results aren’t back yet. Can you ring again between 3 and 4?” – *Patient 1 (male) Practice 1*
    At Practice 2, one of the patients we spoke to still found receptionists had difficulty relaying abnormal results.
    It’s fine if it’s a negative one, but if it’s abnormal it’s hard for them to give the information over the phone. – *Patient 3 (female) Practice 2*
*Teleconsultations*
    To help address patient anxiety engendered when receiving news of abnormal results from receptionists, Practice 2 increased the availability of teleconsultations with FPs. The practice manager commented on this growth.
    We’re definitely seeing an increase in teleconsultations, on average two or three each surgery with each doctor, which I know isn’t a lot, but it’s more than it was and the doctors like it. – *PM (female) Practice 2*
    Patients appreciated the increased availability of their FP via telephone.
    I think it’s a good thing I really do, it’s just a back-up really, just gives you that extra confidence. – *Patient 3 (female) Practice 2*
    For FPs at the same practice the benefit of teleconsultations to them was less apparent.
    The trouble is telephone consultations aren’t quick either, and people say “I thought I’ll talk to you on the phone and then you can save an appointment” and I think we’re not actually saving time. You can be on the phone for seven minutes. Well, the appointments only seven minutes long and they think they’ve done you a favour! – *FP (female) Practice 2*
    The practice manager also conceded that teleconsultations could only offer a limited solution due to constraints on resources.
    Making an appointment a week later for a telephone consultation for your results: we have no problem with that; if everybody did it? You’d sink us, again because we don’t have the capacity for that. – *PM (female) Practice 2*
3. Proactive communication of results
*Standard Messaging Service*
    Staff at both practices had begun to use SMS to communicate results to patients.
    The younger FPs are using it. They’re not using a standardised language. I think it’s an individual thing. So they’re all sort of using it in varying degrees, but all in their own fashion. – *PM (female) Practice 2*
    It’s nice to use a text message as well. – *FP1 (male) Practice 1*
    However the Practice Manager at Practice 2 was still concerned that the patient might be unaware of multiple tests being undertaken on a single blood sample.
    Confidence in its use is growing, but until we can iron out what to do in the case of multiple test results then... – *PM (female) Practice 2*
    For a patient at Practice 1 the concern was if the result prompted a query from an anxious patient there would be a delay before it could be answered.
    I always worry with something like this. How is it going to affect the patients who also suffer with anxiety? There’s no immediate feedback path for someone who suffers with anxiety. – *Patient 1 (female) Practice 2*
*Written notification*
    The letter Practice 2 issues to those with abnormal results, reminds patients of their responsibility to attend appointments. One patient we spoke to at Practice 2 following this advice would book an appointment if a test had been ordered, then either cancel it or, if receiving the letter advising them of an abnormal result, attend the appointment.
    The office manager will always say, “Make an appointment in a week”, and then I either ring up or you’ll get a letter for an abnormal, but you’ve already got an appointment and if you don’t need the appointment you cancel it. – *Patient 3 (female) Practice 2*
4. Increasing awareness of the tests ordered and the mean of communication
  The leaflet containing information on the result ordered that FPs offer to patients during their consultation had only gone live at Practice 1. Here FPs could access and print the form via a link embedded within the SystmOne home page. Initial opinions of staff appeared favourable.
  We talked about it with the entire practice in the big group meeting and yeah, I think it has been positively received by staff. – *FP3 (male) Practice 1*
  It’s great for patients and you can also show them the important things, like whether to phone for results and the number of tests ordered…I think what we all wanted to avoid is this problem, which happens not infrequently, where people ring up and are told “everything is normal”, when in fact not everything is back, and this is the real danger, isn’t it? – *FP2 (female) Practice 1*
  It was pointed out by one FP that if it were to be used more consistently it might require more thorough assimilation into current software.
  It’s an extra click, as simple as that, and it’s not [always] happening for that reason. – *FP1 (male) Practice 1*
  Its use also meant that the FP had to spend some time after presenting the leaflet to patients explaining the contents.
  I found actually it takes an extra minute or two to explain at the consultation, to explain to the patient what I’m actually doing, which I hardly…I haven’t got that time, but I was hoping it was going to help some of the communication issues at the other end, and it probably has helped on occasion. – *FP2 (female) Practice 1*
  At both practices, staff expressed concern that it might be too much paperwork for patients and therefore its continued use would rely on patient uptake.
  I think sometimes the more paperwork, the more work it can be for a patient… you’ve got to be a bit careful, haven’t you? – *Office Manager (female) Practice 2*
  [Its continued use] depends on how many people ring back for results and don’t have the leaflet because it’s completely pointless [otherwise]. I suspect it will be quite a lot of people ringing back and they won’t actually have it in front of them… But it has the potential. If it doesn’t work then we’ll have made the effort and done all we can to make sure they get the right results. – *FP4 (male) Practice 1*
  The patients we spoke to had yet to be offered the leaflet and so were shown examples to gauge their opinion on its design and overall value. Some patients expressed concern that the names of the tests might not be understood by the patient.
  There might be a problem with terminology. I mean, I’ve seen it before, my doctor asked me to have a kidney function test, and it’s on there as ‘the renal profile’ which is, I assume, the same thing? – *Patient 2 (male) Practice*
  Patients liked the fact that it would provide a framework for their discussion with their FP.
  It wouldn’t just be a bit of paper given to you, it would be [the start of] a dialogue: “I’m doing this ‘cause you need that” – *Patient 1 (male) Practice 1*
  Patients at both practices thought it would prove a useful aide-memoire, and appreciated the link to further information on the test.
  I think it’s really good; it would remind those that had a test and so to follow it up really… ‘cause people walk out of the door and they forget. – *Patient 3 (female) Practice 2*
  From my point of view, it’s quite a useful reminder and I would look online to see what the implications of the test are. – *Patient 3 (female) Practice 1*
  Patients also felt it would be a useful reminder of how many tests had been ordered when calling to collect results.
  At least, when you phone through for your results you can say you know what’s missing. – *Patient 2 (male) Practice 1*

## Discussion

### Summary of the main findings

We worked closely with two practices in an attempt to address six key areas of weakness within the testing and results communication process. Despite the use of a collaborative improvement methodology intended to facilitate consensual and applicable improvement strategies, not every suggestion sourced was adopted as prescribed. The different approaches to the same area of weakness in the process were influenced by resource and priority. The larger practice (Practice 1) was able to employ additional staff to ease delays or otherwise possessed the in-house capability to support IT related innovation. In addition differences in staff attitudes meant that only Practice 2 increased the availability of teleconsultations and began to routinely write to patients with abnormal results. However, both increased access to phlebotomy, supported receptionists tasked with relaying clinical information, and conditionally adopted SMS. Of the solutions identified neither practice introduced alerts for missing or delayed tests though the larger Practice 1 failed to do this because of potential changes to their provider of laboratory services and Practice 2 because they did not have the IT capability. Neither practice felt they needed to improve the current systems for handling patient calls.

### Main discussion by area

#### Improved access to phlebotomy

In many countries, same day access to phlebotomy is expected and provided [28], yet both patients and staff at our study practices previously reported regular and frequently lengthy waits for an appointment with a phlebotomist [6, 7]. Some of the failure to match practice resource to patient need can be attributed to the variability of demand inherent in healthcare provision, and can be mitigated by strategic management interventions [29]. In this case both practices were able to reduce the delay in accessing phlebotomy. The more broad adoption of similar improvements across other practices may rely on the routine capture by practices of more precise information on the demand for phlebotomy, current waiting times and a comparative cost assessment of using various staff to provide the service.

#### Improved receptionists’ training

Receptionists already play an essential role in the successful functioning of the surgery and have a major influence on patient satisfaction [30, 31]. The TRaCKED study highlighted the pivotal function they serve in relaying clinically sensitive information [6] despite the lack of any requirement for training or qualifications [32–34]. Both practices acknowledged the need for improved training of receptionists handling results; however, constraints on staff time meant this failed to materialise. It may be that changes are required at a policy level, stipulating precise requirements for training and qualifications in order to raise the priority of reception staff training with senior practice staff [35]. That staff and patients both reported the benefit of the simple guidance that was provided serves to demonstrate that a coherent approach to better supporting receptionists might have considerable benefits.

#### Teleconsultations

To meet patient demand for more responsive feedback on results, Practice 2 increased availability of tele-consultations with FPs. This had the joint benefit of preventing the expense and inconvenience of patients making a further trip to the surgery, and meant they could have any concerns about their result addressed directly by a FP. The patients we spoke to were pleased with this option reflecting the findings of a recent survey in the United States which reported that patients’ preferred method for receiving results was a telephone call from a physician or nurse practitioner [36]. However, though favourably received by patients and one of the PMs, FPs reported how time-consuming it was for them. This has been observed previously and would appear to be a limiting factor in the widespread adoption of teleconsultations [37]. It therefore appears that teleconsultations would only be viable if used selectively.

#### Proactive communication of results

Previous work has found that the use of SMS in communicating test results led to fewer days between diagnosis and treatment [38] and there have been high levels of satisfaction reported among physicians who communicate electronically with patients [39, 40]. The function for FPs to directly text results to patients exists in the clinical management systems used by both practices, yet this was seldom used before our study and during it only by certain FPs. Both practices resisted any more formal adoption of SMS for communicating results amidst concerns about confidentiality and liability for lost results and concerns that patients may be unaware of multiple tests. The greater use of SMS to communicate results in the UK may be a moot point, considering the new contract for NHS mail no longer includes free SMS [41]. Instead, it is likely that other forms of electronic communication may take precedent, including the use of web portals or emails [42].

For Practice 2 the awareness of the potential for error in communicating results motivated them to write to all patients with abnormal results to remind them they needed to attend a consultation with a FP. The concern of senior staff may account for the speed with which the implementation of a potentially costly solution reflects previous research that described how defensive medicine can drive service interventions [43]. One patient we spoke to felt this amendment worked well however previous work in the United States reported that although patients were happy to receive confirmation of normal results via mail, they would prefer notification of abnormal results via telephone where there was opportunity for more instantaneous feedback [44].

#### Increasing awareness of the tests ordered and the mean of communication

Previous studies have demonstrated how providing patients with improved information prior to receiving results can lead to increased levels of reassurance [45]. This information can also help frame ensuing discussions with FPs [46, 47]. Yet previously in participating practices, many patients appeared unaware of exactly which test or tests had been ordered [7], the implications of the test and their role and responsibility in retrieving results [24].

In response we produced an information leaflet that listed the tests ordered, a link to further information on these tests hosted by LabTests Online [48] and the method for result retrieval. The intention was that towards the end of the consultation, FPs would offer the leaflet to patients due to undergo blood tests. The concept proved popular with staff and patients at both practices. However, only the larger Practice 1 that employed a full time IT manager was able to amend the clinical management system to allow FPs to readily print the leaflet. In accordance with previous research [49], the technical nature of this amendment proved challenging for the smaller less well-resourced Practice 2. Instead they were reliant on a software provider that would not alter the functionality of their clinical management system without sufficient popular demand. That this popular support failed to materialise during the time-span of the study is perhaps unsurprising when many practices do not possess the means to audit the successful communication of results and so remain unaware of the potential size of the problem [24].

### Strengths and limitations

The original intention was to provide both quantitative and qualitative evidence of the effect of our improvements on the delivery of results. However current clinical management systems do not possess the functionality to readily capture and collate this information on successfully communicated results and so we relied solely on our qualitative evidence.

We accept that with more time we may have been able to source more individuals that had direct experience of the process improvements we were evaluating. However, the time constraints of a multi-phase study set within busy primary care practices meant we could not wait longer than three months before evaluating the amendments. In addition, it is to be expected that a more concerted and uniform adoption of the proposed solutions did not happen when considering the complications of introducing change into complex microsystems such as primary care practices.

## Conclusions

Though not all of the suggested improvements were adopted, the engagement of practice staff, encouraged by their growing awareness of the problem, meant a significant number of changes to existing systems were usefully implemented. The frequently different ways in which each practice tackled the same issue was influenced by a number of factors including the attitudes of individual staff members, and characteristics of the organisation and its patients.

Our qualitative evaluation found that the changes adopted were well received by both staff and patients. We understand that not every solution we identified, or implemented by our study practices, is applicable to practices elsewhere﻿, yet at the very least this study has helped raise awareness of weaknesses in one of the core functions of primary care. A useful next step would be the collection of dependable empirical evidence of the delays encountered by patients awaiting results and the number of results that either failed to reach practices or failed to be communicated to patients. This may then provide the leverage necessary for the broader uptake of some of the practical solutions adopted here.

## Abbreviations

AS: Administrative staff
FP: Family practitioner
GP: General practitioners
HCA: Health care assistants
IMD: Index of multiple deprivation
PM: Practice manager
PN: Practice nurse
TRaCKED: Test Result Communication Knowledge, Evaluation and Development
